# Extrachromosomal DNA (ecDNA): Unveiling its role in cancer progression and implications for early detection

**DOI:** 10.1016/j.heliyon.2023.e21327

**Published:** 2023-10-29

**Authors:** Shuhong Wu, Tao Tao, Lin Zhang, Xiao Zhu, Xiaorong Zhou

**Affiliations:** a Department of Immunology, School of Medicine, Nantong University, Nantong, China; b Computational Systems Biology Lab (CSBL), The Marine Biomedical Research Institute, Guangdong Medical University, Zhanjiang, China; c Department of Gastroenterology, Zibo Central Hospital, Zibo, China; d Zhejiang Provincial People's Hospital, People's Hospital of Hangzhou Medical College, Hangzhou Medical College, Hangzhou, China

**Keywords:** Amplification of oncogenes, Detection technique of ecDNA, Drug resistance, ecDNA, Carcinogenesis

## Abstract

Extrachromosomal DNA (ecDNA) is a special class of circular DNA in eukaryotes, which is independent of conventional chromosomes. These circular molecules play important roles in biology, especially in cancer biology. The emergence of sequencing technologies such as CCDA-seq and Amplicon Architect has led to a progressive unraveling of the mystery of ecDNA. Consequently, insights into its function and potential applications have begun to surface. Among these studies, the most noteworthy research pertains to cancer-related investigations into ecDNA. Numerous studies have underscored the significance of ecDNA in the pathogenesis of cancer and its role in accelerating cancer evolution. This review provides an overview of the source, structure, and function of ecDNA, while compiling recent advancements in ecDNA in the field of cancer. Nonetheless, further research is imperative to determine its effectiveness and specificity as a biomarker for early cancer detection.

## 1 Introduction

DNA, the genetic material of life, can be divided into two types: linear and circular [1,2]. Eukaryotic genomic chromosomal DNA is found in a linear form, while circular genomic DNA is predominant in organelles such as mitochondria and chloroplasts, as well as in the vast majority of bacteria, and certain viral genomes. Extrachromosomal circular DNA (eccDNA) refers to extrachromosomal, non-mitochondrial, non-chloroplast circular DNA found in eukaryotes. The discovery of eccDNA [3] even precede the widely known mitochondrial DNA [[4], [5], [6]] and bacterial plasmid DNA [7,8], which were confirmed to be circular by microscopic observation in 1966 and 1967 respectively. EccDNA and genomic DNA exhibit significant sequence homology, but they are characterized by substantial differences in size, spanning from hundreds of base pairs (bp) to hundreds of kilobase pairs (KB). Researchers have proposed a variety of names based on this. Specifically, the term “microDNA” mainly refers to circular DNA molecules that are up to 400 bp in length, and the term “ecDNA” [[9], [10], [11]] is used to describe extra-chromosomal DNA found in cancer cells, with sizes reaching hundreds of KB. It is large enough to accommodate full-length genes and DNA replication initiation sites, thus enabling autonomous replication and amplification. This is associated with the amplification of oncogenes [12] and carcinogenesis [13]. EccDNA can carry a complete gene, exhibiting a size range of approximately 1–3 MB. This size parameter remains discernible through examination using a light microscope [14]. As early as 1965, Hotta et al. discovered extrachromosomal circular DNA molecules from wheat embryos and pig sperm [3], and also in the same year, Cox et al. reported the discovery of extrachromosomal DNA molecules in human tumor specimens, which often occur in pairs and are therefore called double-minutes [15]. This is the first time chromosomal-free DNA molecules were observed in vitro in a human tumor. Since then, several studies have found that double microsomes can carry oncogenes, including EGFR and c-Myc, and so on [16]. Subsequent research endeavors have revealed that this extra-pyrocchromosomal DNA molecule often exists in the form of circular DNA [[17], [18], [19], [20]], and further studies have found that only about 30 % of human extra-pyrocchromosomal DNA exists in the form of double microsomes [14]. Therefore, the concept of double microsomes mentioned above is gradually replaced by the expression of extrachromosomal circular DNA (eccDNA). The discovery of ecDNA long predates the completion of the Human Genome Project. As high-throughput sequencing gained prominence and exhibited its efficacy in identifying somatic alterations, the focus of research in the field shifted to genome-wide somatic variation rather than extrachromosomal amplification. Nowadays, as sequencing technology continues to advance, extrachromosomal DNA has been discovered and studied in various fields.

Here, we delve into the intricate world of ecDNA and its intriguing relationship with tumor progression. Our exploration probes into a network of interrelated segments, spanning from fundamental insights regarding ecDNA, including its origin, production, function, structure, and methods of identification and detection. It extends to the tumor-related ecDNA mechanisms involving oncogenes, prognosis, tumor heterogeneity, drug resistance, and molecular markers. As our review traverses these sections, we aim to illuminate the current understanding and potential future directions of this dynamic field, thereby contributing to the broader canvas of cancer biology and paving way for innovative therapeutic interventions.

## 2. Origin, production, and immune stimulation capacity of ecDNA

2

As research into ecDNA has continued to advance, there have been three basic questions about ecDNA for more than half a century: Firstly, whether the sequence homology between eccDNA and genome is specific; Secondly, what is the production mechanism of eccDNA; and thirdly, whether eccDNA has a biological function.

Recently, Zhang et al. [21]combined rolling cycle amplification and the third generation of Oxford Nanopore sequencing technology to obtain the full-length sequence of eccDNA with only third-generation sequencing. The researchers selected eccDNA extracted from mouse embryonic stem cell lines with relatively stable genomes for study and obtained the full-length sequence information and genomic origin location information of more than 1.6 million eccDNA molecules [22,23]. It was found that eccDNA is derived from genomic fragments, including single-fragment self-looping and multi-fragment linked looping [24]. The size of eccDNA ranges from 200bp to 3 kb, exhibiting a distribution pattern similar to the size distribution between oligo-nucleosomes. Apoptosis is a programmed cell death that widely occurs in eukaryotes *in vivo* and cultured cells in vitro [25]. One of its crucial characteristics is the targeted cleavage of genomic DNA by specific nucleases at the internucleosomal region, forming a ladder-like DNA with a regular distribution of oligosomal size [26]. Comparing the eccDNA content of normal cultured cells and apoptotic cells, it was found that the amount of eccDNA in apoptotic cells was significantly increased, indicating that apoptosis can induce the occurrence of eccDNA [24]. The team identified and knocked out DNase1/3, the nuclease responsible for apoptotic DNA fragmentation in mouse stem cells. The intervention led to the development of a cell line with impaired apoptotic DNA fragmentation. Consequently, DNase1/3 KO cell lines also lost the ability of apoptosis-induced eccDNA generation. This indicates that eccDNA occurs from DNA fragments produced by apoptosis. Although it is identified that apoptosis inducers can increase eccDNA generation, which is dependent on apoptotic DNA, the detailed mechanisms are still largely unknown. Till now, there is no direct evidence linking eccDNA to apoptotic markers as far as we know, further research is needed to explore the potential interactions.

The cyclization of linear DNA fragments is dependent on DNA ligase [21]. There are three common DNA ligase genes in eukaryotes including Lig1, Lig3, and Lig4. When Lig1 and Lig4 are depleted, there is no observable impact on eccDNA production. However, in the case of the lig3-deficient cell line, the generation of eccDNA sees a substantial decrease, indicating that Lig3 is the most important ligase responsible for DNA looping [24]. They hypothesized that the circular nature of eccDNA conferred its superior ability to stimulate innate immune responses. In typical immune cells, BMDC (bone marrow-derived dendritic cells) and BMDM (bone marrow-derived macrophages), compared with linear genomic DNA fragments of the same size, their eccDNA can induce higher expression of cytokines such as type I interferon (IFN-α, β), interleukin-6 (IL-6), tumor necrosis factor (TNF-α), etc., showing a superior ability to stimulate immune responses. It is reported that eccDNA enriched with CpG-rich genomic DNA fragments can serve as TLR9 ligands, which increase cell production of IL-6 and TNF-α via activation of the TLR9-MyD88-NF-κB signaling pathway [27]. When the molecular loop of eccDNA is cut off into the corresponding linear molecule, it loses its superior ability to stimulate an immune response, confirming that the strong immune stimulation ability of eccDNA depends on its ring structure [24]. Further studies revealed that the superior innate immune stimulation ability of eccDNA is dependent on the intracellular Sting signaling pathway but not the Myd88 signaling pathway [23].

The above studies have provided direct evidence for the eccDNA puzzle, which has perplexed researchers for more than half a century in three basic directions: 1) EccDNA is randomly derived from chromosomal genomic DNA and has no obvious position or sequence specificity; 2) eccDNA is the cyclization product of Lig3-mediated apoptotic DNA fragments; 3) eccDNA has a superior ability to stimulate innate immune responses. This research [24] has a profound impact on the two fields of apoptosis and innate immunity. It holds particular relevance for numerous conditions characterized by the rapid occurrence of extensive cell death, as well as for disease treatment procedures. Examples of such conditions include acute deadly infectious diseases of bacteria and viruses, immunotherapy of cancer, etc. In addition, this study also provides a new design idea and path for the design of nucleic acid vaccines and vaccine adjuvants. To ensure a comprehensive and systematic approach, the review will follow the guidelines of Preferred Reporting Items for Systematic Reviews and Meta-Analyses (PRISMA), involving a structured process of literature search, study selection, data extraction, and synthesis. The initial search will yield a pool of potentially relevant publications.

## 3 Structure and function of ecDNA

Structure determines function [28,29]. DNA not only stores genetic information in base sequence but also has the capacity to modulate the selective expression of genetic information by altering its higher-order structure. In 2019, Mischel et al. [13]achieved the initial characterization of the structure and function of ecDNA. Deciphering the outcomes related to ecDNA represents the primary stride toward comprehending its functional significance. Paul et al. [13] and his team first combined next-generation whole genome sequencing and optical mapping and found that the amplified ecDNA formed a circular structure from the sequence perspective. Using RNA-Seq technology combined with single-nucleotide polymorphism analysis, researchers found that circular ecDNA in tumors can also direct gene transcription. In tumors, the predominant oncogene transcripts are directly derived from ecDNA, since ecDNA tends to have higher copy numbers (even than those amplified in chromosomes) and carry oncogenes. They noticed that the chromatin of ecDNA is highly open, providing a deeper explanation for the abundant expression of oncogenes on ecDNA. The three-dimensional structure of chromatin enables the interactions between DNA elements to occur [30]. As far as we know, this study marks the initial endeavor in unraveling the circular structure and function of tumor extra-chromosome DNA. It is also clarified that ecDNA is circular, and chromatin is highly open, and ecDNA can mediate ultra-long-distance interactions due to its circular structure [31,32]. The results of this study reveal the function of ecDNA in expressing a large number of oncogenes and further explain the reason for the abundant expression of oncogenes on ecDNA.

## 4 Identification and detecting technique of ecDNA

In this section, we have provided an all-encompassing overview of the methodologies harnessed for the identification and detection of ecDNA, shedding light on its elusive presence and characteristics. We first dissected the intricate details of ecDNA identification, where we delved into cutting-edge methods like Amplicon Architect and embarked on an exploration of ecDNA junction site recognitions. Besides, we also ventured into the field of chromatin openness with CCDA-seq, a novel technique unraveling ecDNA features at a single molecule level. We also discuss sequencing methods tailored for ecDNA, including tissue cell circular DNA sequencing, serum, and plasma circular DNA sequencing, and circular DNA methylation sequencing.

### 4.1 The identification of ecDNA

#### 4.1.1 Amplicon Architect

Amplicon Architect [33] was developed for identifying and analyzing the fine structure of amplicons. This tool has successfully pinpointed a substantial array of human-viral heterozygous extrchromatin Amplicon structures within diverse tumor types. The methodology amalgamates the utilization of metaphase fluorescence in situ hybridization (FISH) [34] and in conjunction with PacBio sequencing, thereby achieving comprehensive analysis of these structures. The fine structure of an extrachromosomal heterozygous amplicon containing FOXE1 (Forkhead box E1) in the UPCI: SCC090 cell line was mapped (Table 1). The principle of Amplicon Architect is based on two features of ecDNA: 1, The copy number was significantly amplified [34]. 2, It is a special ring structural variation (SV) [13]. Amplicon Architect is a tool that uses whole-genome sequencing data to reconstruct ecDNA Amplicon structures. Amplicon Architect provides a universal Amplicon model encoding all supporting structures, as well as an algorithmic framework for reconstructing the possible structures of amplicons (Table 1）, thereby restoring the authentic eccDNA structure as much as possible.

**Table 1:** The latest methods for the detection of ecDNA.

method	Recognition site	Significance	references
Amplicon Architect	Extrachromosomal amplicon structure	AA provides a generic amplicon model encoding all supporting structures, while providing an algorithmic framework for reconstructing the possible structures of amplicons.	[33]
Circle-Map	EcDNA junction site	Circle-map uses posterior probability to re-align the soft splice part with the reference genome and then reconstruct it into circular DNA breakpoint Map, which can more accurately detect circular DNA break points.	[35]
Circle-finder	EcDNA junction site	Circle-finder uses existing NGS data analysis software and can analyze ecDNA fragments in ATAC-seq data by concatenating them with shell scripts.	[36]
ECCsplorer	EcDNA junction site	The eccDNA candidate genes thus observed by ECCsplorer can be further exploited for a wider range of research, from tracing active transposable elements to therapeutic approaches for cancer-related ecDNA.	[37]
CCDA-seq	EcDNA junction site	CCDA-seq was used to observe the diversity of ecDNA in open chromatin regions, revealing the different chromatin states of linear DNA and ecDNA at the single-molecule level.	[44]

#### 4.1.2 Recognition of ecDNA junction sites

##### 4.1.2.1 Circle-Map

Circle-map [35] takes as input the alignment of the read sequence to the reference genome, which is used by Circle-Map to detect the splitting of the read into two parts to detect genomic rearrangements supporting the circular DNA structure. However, Circle-MAP can result in the loss of numerous split alignments [35], primarily due to the split segment being excessively short or the sequence identity being so strong that it cannot align to multiple positions (more than two). All these alignments were defined as soft-clipping. Circle-map uses posterior probability to re-align the softly spliced part with the reference genome, subsequently reconstructing it into a circular DNA breakpoint Map. This refined approach enhances the accuracy detecting circular DNA junction sites (Table 1).

##### 4.1.2.2 Circle_finder

Circle_finder [36] uses existing NGS data analysis tool [33,[35], [36], [37]] and it can analyze ecDNA fragments in ATAC-seq data by concatenating them with shell scripts (Table 1). The core algorithms include the use of Samblaster software to distinguish between discontinuous alignments and split alignments, and BEDtools to deal with genome interval problems.

##### 4.1.2.3 ECCsplorer

ECCsplorer [37] is a bioinformatics pipeline for the automatic detection of eccDNA from double-end sequencing data of amplified circular DNA. ECCsplorer primarily operates through two distinct steps [37]: 1. Alignment of sequences to the reference genome, and then detection of abnormal alignment distribution, including High coverage, discordant reads and Split reads. 2. The no-reference set of amplified eccDNA is compared with a control sample that is determined to be specifically enriched. The observed eccDNA candidates can be further exploited for a wider range of research from tracing active transposable elements to developing therapeutic approaches for cancer-related eccDNAs (Table 1). Compared with Circle-Map and Amplicon Architect, this process exhibits a higher level of integration and better presentation of results.

### 4.2 CCDA-seq for studying open chromatin structure of ecDNA

Current studies of ecDNA have focused on identifying ecDNA from sequencing data analysis using copy-number variants (e.g., Amplicon Architect) [33]and detecting ecDNA junction sites (e.g., Circle-MAP and CIRC_finder) [35,38]. When studying ecDNA with traditional methods such as ATAC-seq [39] and chip-seq [40], DNA is interrupted and short fragments are sequenced. Most of the computational methods used aim to identify ecDNA by detecting junction sites. Therefore, they can only reveal the chromatin accessibility and modifications of DNA-binding protein in the adjacent junction sites, However, they fall short of offering a comprehensive depiction of the complete ecDNA chromatin landscape and fail to elucidate the interplay between regulatory elements located in middle and distal regions. In addition, traditional data analysis based on peak calling strategy cannot provide single-molecule ecDNA structure and epigenetic information. Consequently, it falls short in capturing the diverse ecDNA structures and epigenetic profiles across distinct states, precluding the characterization of epigenetic diversity inherent in ecDNA molecules across various states.

In the past two years, novel technologies such as nanoNOMe-Seq [41], SMAC-seq [42], and Fiber-seq [43] have emerged, which are developed based on single-molecule long-read sequencing methods. These innovative methods allow for the concurrent acquisition of both nucleotide sequence and methylation information of a single DNA molecule. They have been found to apply in the aspects of single-molecule epigenetic heterogeneity and distal interaction of regulatory elements. However, it has not been reported in the field of ecDNA.

In 2021, Chong Tang et al. [44] used m6A MTase methyltransferase to treat genomic DNA, enabling the acquisition of m6A DNA methylation modifications of open chromatin regions at the single-molecule level. Based on the position of the junction, part of the ecDNA sequence was reconstituted as a new reference, and the m6A signal was identified according to the reconstituted ecDNA sequence to prevent signal bias in the junction region, and the complete ecDNA was reconstructed. The DNase-seq data showed similar nucleosome patterns around the transcription start sites of highly expressed genes, which were also highly consistent with the genome scale at different resolutions. The sequenced reads were between 10 and 100 kb in length, which is about 50 times wider than the junction regions observed in conventional ATAC-seq [39]. Through a subsequent comparison of average chromatin accessibility between ecDNA and its homologous linear DNA, the researchers observed a prevalent trend wherein the majority of ecDNA exhibited significantly heightened chromatin openness, surpassing that of linear DNA. This observation further bolsters the overarching notion that ecDNA amplification leads to higher oncogene transcription. RNA-seq data analysis showed 340 ecDNA highly expressed genes (25%rank) and 464 moderately expressed genes (75%-100%rank), indicating that not all ecDNA genes are highly expressed [44]. By analyzing chromatin accessibility signals near transcription start/stop sites, CCDA-seq effectively identifies regions characterized by nucleosome depletion, commonly known as nucleosome depletion regions (NDRS).

CCDA-seq provides chromatin states with single-molecule resolution, spanning thousands of base pairs in length. By analyzing and comparing the distribution of chromatin signals at the single-molecule level in ecDNA and linear genomic DNA, we observed multiple chromatin accessibility states in linear DNA and ecDNA. Linear DNA (chr10: 4238321–42389251) exists in two different conformations: one is a nucleosome-inactive state, and the other is a state that lacks nucleosomes due to extremely high transcriptional activity, while a highly heterogeneous nucleosome depletion/occupancy pattern is observed in ecDNA, and the apparent heterogeneity of molecules in different states cannot be obtained in bulk ATAC-seq [44].

In summary, CCDA-seq was used to observe the diversity of ecDNA in open chromatin regions, localize the distribution of nucleosomes at the kilobase length scale, and quantify the chromatin state correlation of distal regulatory elements at single-molecule resolution (Table 1). This method unveiled distinct the chromatin state of linear DNA and ecDNA at the single-molecule level. CCDA-seq contributes to a more comprehensive understanding of the regulation of the ecDNA epigenome and provides valuable insights into the unique mechanisms of ecDNA regulation.

### 4.3 Sequencing methods for ecDNA

#### 4.3.1 Tissue cell circular DNA sequencing

The experimental process of tissue cell circular DNA sequencing [45] can be divided into four steps, which are 1. Column purification; 2. Enzyme digestion; 3. rolling ring amplification; 4. Library construction and sequencing. Given that the amount of circular DNA within cells is small relative to the abundant genomic DNA, the initial step following the total DNA extraction from the sample involves, removing the genomic DNA by column purification. This ensures that the genomic DNA does not occupy the majority of sequencing data volume. Column purification is a critical step to maximize the removal of genomic DNA and the retention of circular DNA molecules, especially to avoid the loss of ultra-long and ultra-short circular DNA. Even after column purification, a residual amount of linear DNA might persist. Therefore, in the second step, circular DNA's resistance to restriction endonucleases is exploited, and exonucleases are employed to digest the product purified by the column. For human samples, circular mitochondrial DNA is cleaved into linear DNA using the restriction enzyme Mssl, which is specific for human mitochondria, followed by exonuclease digestion to remove mitochondrial DNA. Following the initial two steps of enzymatic digestion and column purification, linear genomic DNA is removed, leaving behind a small quantity of circular DNA. To attain the necessary DNA quantity for library construction, it becomes imperative to amplify the circular DNA signal by rolling circle amplification. After purification and amplification, the enriched circular DNA was subjected to routine NGS high-throughput sequencing. The workflow includes fragmentation, library construction and 150-bp double-end sequencing using the Illumina NovaSeq sequencer.

#### 4.3.2 Serum and plasma circular DNA sequencing

The experimental process of serum and plasma circular DNA sequencing [46] can be roughly divided into four steps, which are: 1. Circular DNA enrichment; 2. Add connectors; 3. End repair; 4. Library construction and sequencing. Initially, the linear DNA in the sample is removed by exonuclease V digestion and other means, achieving the purpose of enriching circular DNA. Next, the circular structure of the circular DNA is opened by the transposase, and connectors are added to both ends of the DNA fragment. Finally, the end gap of the transposition product is repaired by the Klenow enzyme, and the purified library is sequenced after qualified quality inspection. This technique offers several advantages, including minimized DNA loss, preservation of the original expression profile, and enhanced accuracy in comparing expressions among different circular DNAs.

#### 4.3.3 Serum and plasma circular DNA methylation sequencing

The experimental process of serum and plasma circular DNA methylation sequencing [47] can be divided into six steps, which are: 1. Circular DNA enrichment; 2. add connectors; 3. end repair; 4. C–U transformation; 5. PCR amplification; 6. Sequencing. Above all, the linear DNA in the sample is removed by exonuclease V digestion and other means, thus enriching circular DNA. Then the circular structure of the circular DNA is opened by the transposase, and connectors are added to both ends of the DNA fragment. The terminal Nick of the transposition product is repaired by the Klenow enzyme again, and the unmethylated cytosine (C) is efficiently converted to uracil (U) by gentle enzymatic conversion. In the end, the transformed products are amplified by PCR and purified, and the purified library is sequenced following qualified quality inspection. The advantage of this technology lies in its capacity to simultaneously detects circular DNA and its corresponding methylation status, a feature that conserves samples and proves cost-effective.

## 5 Mechanisms of ecDNA involved in tumor carcinogenesis progression, heterogeneity, and evolution

This section aims to unravel the intricate interplay between ecDNA and the multifaceted landscape of tumor progression. We first reveal the vital role of ecDNA as a potent amplifier of oncogenes, delving into its role in chromatin opening, oncogene expression, and gene transcription. Building upon this foundation, we describe the association among ecDNA, oncogene amplification, and poor prognostic outcomes across diverse cancers. Continuing our investigation, we delve deeper into the intricate interplay among ecDNA, tumor heterogeneity and drug resistance. Additionally, we underscore the potential significance of ecDNA as a tumor molecular marker, presenting promising avenues for diagnostic and prognostic applications. Finally, we venture into the realm of ecDNA mutations in the evolution and prognosis of cancer.

### 5.1 EcDNA promotes the amplification of oncogenes

#### 5.1.1 EcDNA promotes chromatin opening and oncogene expression

In 2019, the structure of extrachromosomal DNA and the characteristics of high expression of oncogenes were clarified in tumors for the first time [13]. However, as early as May 2019, a review introducing the process of rediscovery of oncogene amplification on ecDNA was published, emphasizing the importance of ecDNA in tumor pathogenesis and accelerating cancer evolution [14]. Extra-chromosomal DNA (ecDNA) is ubiquitous in human cancers [34], and it mediates oncogene overexpression through amplification and alteration of gene regulatory mechanisms [13]. Gene expression induction typically involves contacting and activating *cis*-regulatory elements of genes on the same chromosome. Hung et al. [48] found that clusters of approximately 10–100 ecDNAs contributed to intermolecular enhancer-gene interactions in the nucleus and overexpression of proto-oncogenes.

EcDNA encoding a variety of different proto-oncogenes forms hubs in a variety of cancer cell types and primary tumors. When clustered with other ecDNA, each ecDNA is more likely to transcribe an oncogene. In colorectal cancer cell lines with MYC amplification [49], ecDNA forms a hub through its interaction with bromodomain and BET protein BRD4 [48], BET inhibitor JQ1 can break up the ecDNA hub and preferentially inhibit the transcription of ecDNA-derived oncogenes [48]. The PVT1 promoter that binds BRD4 is ectopically fused to MYC and amplified in ecDNA, and this fusion promoter drives efficient expression of MYC [48]. In addition, the PVT1 promoter on an exogenous fragment is sufficient to mediate *trans*-gene activation at the ecDNA hub, a process that is sensitive to JQ1. Conducting global level silencing of ecDNA enhancers by CRISPRi exposed intermolecular interactions between enhancers and genes, resulting in the activation of multiple amplified proto-oncogene loci distributed across different ecDNAs. Protein-linked ecDNA hubs enable intermolecular transcriptional regulation and may serve as units of oncogene function and cooperative evolution, as well as potential targets for cancer therapy (Table 2).

**Table 2:** Discovery of ecDNA in cancer research.

Biological effects	The research methods	Significance	References
EcDNA promotes pro-oncogene expression	Global level silencing of ecDNA enhancers by CRISPRi.	Protein-linked ecDNA hubs enable intermolecular transcriptional regulation and may serve as units of oncogene function and cooperative evolution, as well as potential targets for cancer therapy.	[48]
EcDNA regulates gene transcription	By detecting the dynamic changes in ecDNA space, expression and transcription level of MYc-PVT1 fusion gene.	It has important implications on how oncogene regulation on ecDNA contributes to oncogene transcription and oncogene heterogeneity.	[50]
EcDNA promotes tumor drug resistance	Direct observation of chromosome structure and whole-genome sequencing of drug-resistant cells.	The higher the level of gene mutation in EcDNA, the higher the heterogeneity of tumor cells and the stronger the drug resistance of tumor cells. At the same time, there is a highly specific, dynamic and adaptive pathway in tumor cells that promotes tumor cell drug resistance.	[61]
EcDNA promotes genetic recombination in cancer cells	To explore the role of chromosomal circular DNA in neuroblastoma by combining genomics and transcriptomics.	This study identified a major source of somatic genome rearrangement by extrapymosomal circular DNA and revealed that extrapymosomal circular DNA can actively drive genome rearrangement and promote aberrant cancer-related gene expression patterns.	[56]
EcDNA can be used as a tumor molecular marker	Circle-seq was used to isolate and identify ecDNA.	The presence of ecDNA in the blood of cancer patients, and the isolation and identification of ecDNA can monitor the characteristics and mutations of tumors in real time.	[64]

Future studies may identify proteins that mediate ecDNA transcriptional activity in various types of cancer, yielding crucial insights instrumental in formulating new avenues for therapeutic interventions.

#### 5.1.2 EcDNA regulates gene transcription

**Fig. 1:**
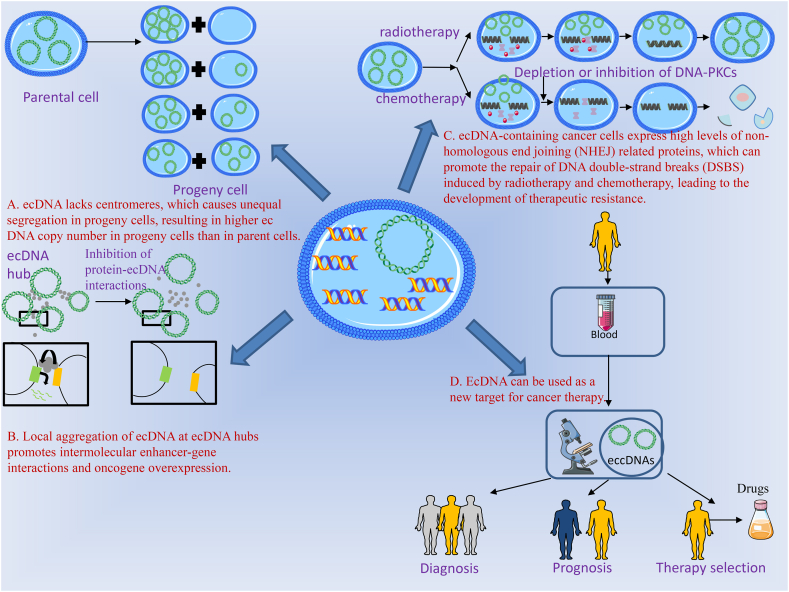

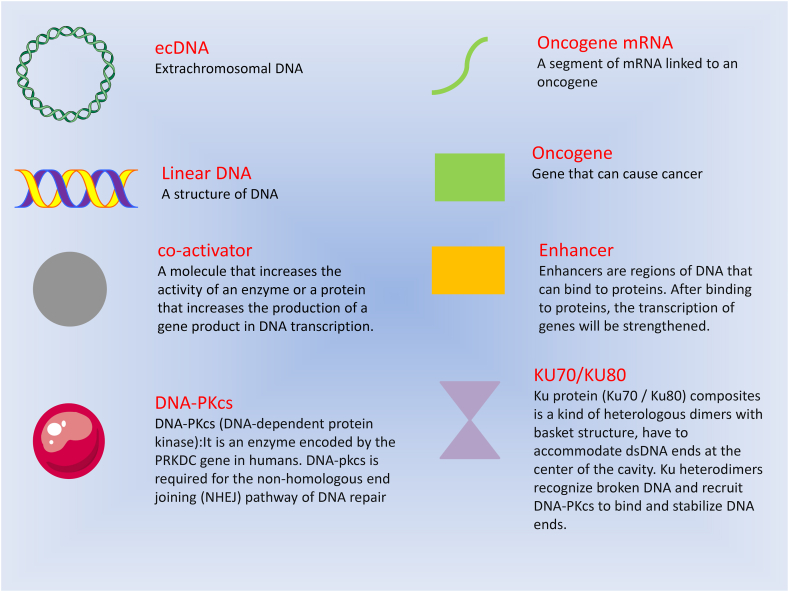
(a)Discovery of ecDNA in tumor-related studies. This diagram shows four mechanisms of ecDNA in tumors. **A.** Due to the lack of centromeres, ecDNA was unevenly separated in progeny cells, which resulted in a higher ecDNA copy number in progeny cells than that in parental cells, indicating the mechanism by which ecDNA facilitates the expression of oncogenes.(b). The local aggregation of ecDNA at ecDNA hubs promoted intermolecular enhancer gene interaction and oncogene overexpression, indicating that ecDNA hubs promote oncogene transcription. **C.** EcDNA-containing cancer cells express higher levels of non-homologous end-joining related proteins. This heightened expression promotes the repair of ecDNA double-strand breaks induced by radiotherapy and chemotherapy, consequently driving the occurrence of therapeutic resistance. This mechanism underscores how ecDNA contributes to the resistance of therapeutic agents employed in cancer treatment. D. With the discovery of the related mechanism of ecDNA in cancer, and due to the ubiquitous presence of ecDNA in cancer, targeting ecDNA may become a major direction of clinical treatment of cancer in the future.

Hung et al. [48] observed the evolving alterations in ecDNA involving the MYc-PVT1 fusion gene across spatial, epigenetic, and transcriptional tiers. They identified that ecDNA hubs act as enhanced subsets of the combination (Table 2), demonstrating their regulatory function in the transcription of oncogenes(Fig. 1）. This study reveals that ecDNA-carrying oncogenes appears in clusters in cancer cells, forming ecDNA hubs. Such ecDNA hubs promote novel enhancer-promoter interactions and oncogene expression. In addition, ecDNA hubs may also involve transcription regulatory elements on ecDNA molecules, which may also regulate gene expression at long distances. This finding holds significant consequences for understanding how the control of oncogenes on ecDNA influences both oncogene transcription and cancer cell heterogeneity. CHIA-PET and CHIA-drop chromatin interaction assays were used to explore eccDNA-mediated chromosomal interactions affecting transcription processes in cancer cells at the genome-wide level [50]. In this study, we found that ecDNAs in cultured glioblastoma patient-derived neurospheres and prostate cancer cells had extensive ecDNA-ecDNA interchromosomal interactions. EcDNA-chromosome interaction sites have extensive and high levels of H3K27ac signaling, which are mainly concentrated on chromosomal genes with increased expression levels. The addition of synthetic DNA containing characteristic enhancers to prostate cancer cells results in genome-wide activation of chromosomal gene transcription. Analysis of the chromosomal targets of ecDNAs at single-molecule resolution revealed that actively expressed oncogenes were spatially clustered in ecDNA-mediated interaction networks. These findings underscore the role of ecDNAs as mobile transcriptional enhancers, actively fostering tumor development. Additionally, the results shed light on a potential mechanism of transcriptional regulation implicated in aneuploidy synthesis (Table 2).

### 5.2 EcDNA is associated with the amplification of oncogenes and poor prognosis of various cancers

EcDNA amplification occurs frequently in most cancer types other than hematologic cancers [51]. Oncogene amplification is highly enriched on ecDNA, and the most common recurrent oncogene amplification is also present on ecDNA. This study shows that the prevalence of oncogene amplification through ecDNAs is widespread in various cancers. Unlike chromosomal amplification, this phenomenon is associated with unfavorable prognoses across multiple cancer categories, highlighting the potential role of ecDNA in tumor invasion and the possibility of using ecDNA for prognostic research. Research findings have indicated a substantial prevalence of ecDNA in colorectal cancer (CRC). Furthermore, among CRC patients, those whose cancer cells exhibit ecDNA have demonstrated shorter survival than others [52]. Burgeoning studies have also demonstrated an increase in the frequency of ecDNA between Barrett's esophagus-associated early esophageal adenocarcinoma (24 %) and stage esophageal adenocarcinoma (43 %), indicating that ecDNA formation occurs during the progression of cancer [2]. The expression of microRNA-17-92 and its corresponding microRNA in hepatocellular carcinoma (HCC) tumors is upregulated, which is significantly associated with unfavorable outcomes and patient age [53]. ecDNAs can also be detected across a wide range of cancer types, including breast cancer, glioblastoma, and ovarian cancer [54].

Chang et al. [48] found that ecDNA aggregated in interphase nuclei can act in clusters, driving intermolecular enhancer signals to amplify oncogene expression. This study shows that ecDNA "bunging behavior" promotes novel intermolecular enhancer-gene interactions and oncogene overexpression, which can occur in different tumor loci and multiple oncogene overexpression, as well as in different tumor loci and multiple cancer types. Unlike conventional *cis*-chromosomal transcription, ecDNA central *trans*-regulation enables intermolecular transcriptional regulation, This revelation holds significant implications for advancing our understanding of optimizing oncogene transcription processes. A major mechanism [55] was identified that triggers local amplification of genomic DNA in the form of ecDNA upon chromosomal fragmentation in specific HeLa cells that are resistant to methotrexate. This study offers a fresh insight into the rapid adaptation of cancer cells to new growth conditions and the development of drug tolerance. Future studies may unveil the specific proteins responsible for regulating the transcriptional activity of ecDNA in diverse types of cancer. These proteins could hold promise as viable targets for therapeutic interventions. As we look ahead, the landscape of cancer research is poised to expand into novel domains (Table 2).

### 5.3 EcDNA drives oncogenic genome remodeling in neuroblastoma

David et al. [56] combined genomics and transcriptomics to explore the role of chromosomal circular DNA in neuroblastoma and found a variety of extra pyro chromosomal circular DNA that had not been discovered before. It has also been found that extrachromosomal circular DNA is the main source of somatic genome rearrangement (Table 2). Circular DNA exhibits the ability to undergo chimerism, reintegrating into the linear genome, and promoting the remodeling of oncogenes. This study reveals that extrachromosomal circular DNA can actively drive genomic rearrangements and promote aberrant tumor-associated gene expression patterns. This study has been limited to neuroblastoma and warrants extension to encompass other tumor types in subsequent research. Additionally, it offers novel insights that can guide the exploration of alternative roles played by extrachromosomal circular DNA.

### 5.4 EcDNA promotes tumor heterogeneity and drug resistance

#### 5.4.1 EcDNA promotes tumor cell evolution and genetic heterogeneity

Short fragments of circulating DNA encoding cancer genes were found that may be common in cancer cells. These fragments play an important role in generating the cellular diversity, a factor that contributes to the challenging treatment of malignant cancers [34]. This study shows that tumors become diverse or heterogeneous when oncogenes are amplified in ecDNA, which allows tumors to complete and maintain high levels of oncogene expression. Tumor cells are different from chromosomes in that when tumor cells divide, ecDNA will randomly enter the offspring cells, so any given cell in a tumor may not contain ecDNA in the nucleus. Noteworthily, the higher the level of gene mutations in ecDNA, the higher the heterogeneity of cells in a tumor [34]. This cellular diversity can foster increased resilience of tumors towards external challenges, especially drug therapies (Table 2).

#### 5.4.2 EcDNA promotes drug resistance in glioblastoma

EcDNA plays a central role in drug resistance in glioblastoma [57]. The team found that glioblastoma cells can develop resistance to the epidermal growth factor receptor (EGFR) -targeting drug erlotinib by eliminating an extrachromosomal copy of the mutated EGFR gene. The results of this study suggest a highly specific, dynamic, and adaptive pathway in tumor cells, enabling cancer cells to circumvent oncogene therapy targeting ecDNA.

#### 5.4.3 Chromosome fragmentation fosters ecDNA formation, driving cancer cell drug resistance

Previous studies have shown that chromosome fragmentation is associated with a variety of cancers and congenital diseases [[58], [59], [60]]. Chromosome fragmentation accelerates the rearrangement and amplification of genomic DNA into extrachromosomal DNA, allowing cells to rapidly acquire resistance to changing growth conditions and thus confer resistance to cancer therapy [61]. During the course of chromosome fragmentation, a segment of the chromosome is retained as ecDNA, while a subset of ecDNA elements forms double microsomes [61]. Double microsomes commonly bear amplifications of oncogene and drug-resistance gene, which is closely related to genomic instability, the malignant nature of tumors, and the development of drug resistance [62]. Chromosome fragmentation can convert intrachromosomal amplification to extrachromosomal amplification. The resultant expanded ecDNA responds to DNA damage caused by cancer treatments such as chemotherapy or radiotherapy. This ecDNA then undergoes reintegration into novel chromosomal sites. This study confirms that chromosome fragmentation is the driving force of drug resistance and DNA repair pathways in cancer cells. Furthermore, these findings propose a strategic avenue for clinicians to prudently design drug combinations, aiming to preempt the development of drug resistance, and thus enhance the therapeutic efficacy for individuals afflicted with cancer（Fig. 1）.

### 5.5 EcDNA has the potential as a tumor molecular marker

EcDNA present in the blood of cancer patients could be isolated and identified by liquid biopsy using Circa-seq [63] (Table 2), allowing real-time monitoring of tumor characteristics and mutations. EccDNA can be used as a potential biomarker for many diseases related to mutations and genomic rearrangements [64]. EccDNA from T-cell receptor genes has demonstrated its utility in the detection of severe combined immunodeficiency diseases. The presence of small extrachromosomal circular DNA (eccDNA) derived from specific genes in tissue and plasma holds significant diagnostic value across various types of cancers, particularly enhancing the precision of CEA/CA19-9 levels in predicting diverse cancer types. Small extrachromosomal circular DNA (eccDNA) can serve as an optimal biomarker for cost-effective diagnosis and surveillance of multiple cancers [65]. The extrachromosomal circular DNA (ecDNA) can serve as a promising therapeutic target and non-invasive biomarker for prenatal diagnosis, early detection, prognosis, and treatment of gynecological malignancies [66]. EcDNA in the biallelic form harboring MYCN might be regarded as a potential biomarker for establishing clinical risk assessment in neuroblastoma patients [56,62,64] (Fig. 1）.

### 5.6 Mutations in ecDNA reveal the evolution of cancer and its prognosis

Clustered somatic mutations have been an understudied area of cancer development. Alexandrov et al. [67] identified mutation clusters in certain regions of the genome as previously unrecognized key factors in cancer evolution. Notably, these mutation clusters contribute to the development of approximately 10 % of human cancers and can be used to predict patient survival. The APOBEC3 enzyme is an antiviral factor typically present within cells as a component of their intrinsic immune defense mechanism [68], but in cancer cells, the research team claims that the APOBEC3 enzyme might have deleterious effects instead of beneficial ones. The team reveals that cancer cells with extrachromosomal DNA loops containing known cancer driver genes exhibited a sequence of mutations within ecDNA molecule. The team attributed these mutations to APOBEC3 enzyme activity. These researchers hypothesized that the APOBEC3 enzyme considered the circles of ecDNA as foreign viruses, prompting attempts to restrict and cut them. This enzymatic activity subsequently triggers the emergence of mutation clusters within a single ecDNA molecule.

This, in turn, plays a key role in accelerating cancer evolution and potentially leading to drug-resistance mutations. This signifies a wholly novel mode of tumorigenesis. In conjunction with targeting ecDNA, clinicians can also contemplate the potential approach of restraining the activity of the APOBEC3 enzyme as part of future cancer therapies.

## 6 Conclusion, challenges, and perspectives

This comprehensive review delves into the multifaceted role of ecDNA within the realm of cancer-related research. It encompasses various dimensions including the origins of ecDNA, its intricate structural features, its diverse array of functions, and the latest advancements in sequencing methodologies. The circular architecture of ecDNA, originating from genomic chromosomal DNA through Lig3-mediated apoptotic DNA fragments, imparts it with a distinct ability to engage innate immunity responses. This distinctive attribute underscores its potential significance in apoptosis and innate immunity studies. Although the origin of ecDNA has been preliminarily understood, its specific mechanism still needs to be further studied. At present, a hypothesis has been proposed that a large proportion of eccDNA molecules contain or are close to short direct repeats, suggesting that some form of microhomology-directed repair may form eccDNA. The verification of this hypothesis necessitates further in-depth exploration and investigation.

EcDNA's unique capacity to mediate ultra-long-distance interactions extends its functional repertoire, opening doors to a myriad of biological functions that warrant exploration. Of note, the propensity of ecDNA to facilitate chromatin opening paves the way for robust oncogene expression, thus solidifying its involvement in cancer development and driving the evolutionary trajectory of malignancies. This revelation provides a strategic foothold for novel therapeutic directions, including the intriguing prospect of targeting ecDNA in the pursuit of refined tumor treatments, a concept intricately intertwined with the realm of targeted therapies.

As ecDNA research continues to deepen, it emerges as a potent enigma, offering tantalizing glimpses into a future marked by refined diagnostic accuracy, personalized therapeutic interventions, and ultimately improved patient outcomes. Of course, it is admitted that ecDNA-related research is still in its preliminary stage. There remains a considerable journey ahead to comprehensively unravel its underlying mechanisms of action and to effectively implement it across various clinical domains, especially in the treatment of tumors. The concerted endeavors of scientists are poised to benefit more patients.

## Data availability statement

No data was used for the research described in the article.

## Funding

This study was partially supported by the 10.13039/501100001809National Natural Science Foundation of China (32170915 and 82172931).

## Ethical approval and consent to participate

Not applicable.

## CRediT authorship contribution statement

**Shuhong Wu:** Writing – original draft, Writing – review & editing. **Tao Tao:** Writing – original draft, Writing – review & editing. **Lin Zhang:** Investigation. **Xiao Zhu:** Supervision, Writing – review & editing. **Xiaorong Zhou:** Funding acquisition, Supervision, Writing – review & editing.

## Declaration of competing interest

The authors declare that they have no known competing financial interests or personal relationships that could have appeared to influence the work reported in this paper.
